# Automation in cell and gene therapy manufacturing: from past to future

**DOI:** 10.1007/s10529-019-02732-z

**Published:** 2019-09-20

**Authors:** P. Moutsatsou, J. Ochs, R. H. Schmitt, C. J. Hewitt, M. P. Hanga

**Affiliations:** 1 School of Life and Health Sciences, Aston University, Aston Triangle, Birmingham, B7 4ET UK; 2 Fraunhofer Institut für Produktionstechnologie IPT, Steinbachstrasse 17, 52074 Aachen, Germany; 3 Laboratory for Machine Tools and Production Engineering (WZL), RWTH, Aachen, Germany

**Keywords:** Automation, Bioreactors, Cell therapy, Gene therapy, Manufacturing

## Abstract

As more and more cell and gene therapies are being developed and with the increasing number of regulatory approvals being obtained, there is an emerging and pressing need for industrial translation. Process efficiency, associated cost drivers and regulatory requirements are issues that need to be addressed before industrialisation of cell and gene therapies can be established. Automation has the potential to address these issues and pave the way towards commercialisation and mass production as it has been the case for ‘classical’ production industries. This review provides an insight into how automation can help address the manufacturing issues arising from the development of large-scale manufacturing processes for modern cell and gene therapy. The existing automated technologies with applicability in cell and gene therapy manufacturing are summarized and evaluated here.

## Introduction

Cell and gene therapies are medicinal products that utilise cells and genes to treat disease. These new therapeutics are the next frontier of medicine with unlimited potential, but also with numerous challenges still to overcome until affordable and safer products are available.

According to the database of the US National Library of Medicine (US Department of Health & Human Services 2017), there are currently more than 33,000 cell and gene therapy clinical trials worldwide to date, either ongoing or completed. However, only a small proportion of these (12 in Europe and 17 in US) have received marketing approval to date (EMA 2018; US Food and Drug Administration 2017). This could be due to many challenges related to manufacturing that are still to be addressed. Marketing and regulatory approval of advanced therapies have two main requirements: (1) to demonstrate the safety and efficacy of the therapy in treating the targeted disease (de Wilde et al. 2018) and (2) to demonstrate consistent and rigorous manufacturing to a well-defined product quality (Morrow et al. 2017). Automation has the potential to address many of these existing challenges, while facilitating the requirements for regulatory and marketing approval. This review will cover the current requirements for manufacturing cell and gene therapies, the added value of automation and the existing automated technologies.

## Cell and gene therapy manufacturing requirements

The current manufacturing processes for cell and gene therapies are largely manual, mostly performed in planar culture systems. These are highly laborious, often involve open processes which are difficult to scale-up and rely heavily on the operator’s experience and judgement. As a consequence, they are prone to human error and they can result in increased batch-to-batch variability, high manufacturing costs, increased risk of contamination and batch loss.

Cell and gene therapies typically rely on patient or donor cells as starting material for their manufacture which determines significant batch-to-batch variation inherent to the complexity of the biological product (Heathman et al. 2016). When manufacturing such highly complex products, it is highly important to acknowledge that any change, regardless of how minor in the culture environment as determined by the manufacturing process may result in the alteration of product quality which is a key determinant of its safety and efficacy (Morrow et al. 2017). In order to comply with these requirements, it is suggested that the manufacturing process should be simple enough to allow reproducibility and of a short duration to minimise costs associated with resources and labour (Masri et al. 2017).

What is more is that cell and gene therapy products are not only highly complex, but at the same time, they have to comply with the strict regulatory framework. There is a requirement that these cell-based products are produced in accordance with good manufacturing practise (GMP) which will minimise process variability and therefore variation in cell quality. GMP compliance can be achieved when consistent GMP-grade materials from well-characterised sources are utilised (Medicine Manufacturing Industry Partnership 2016). However full GMP-compliance in the cell and gene therapy realm is currently challenging, particularly because of the increased difficulty in sourcing compliant starting material. This challenge is exacerbated when taking into account the variability associated with manufacturing patient-specific therapies (autologous) where the donor is the diseased patient which means that cell quality will not be of the required standard.

In contrast to ‘traditional’ biopharmaceutical production where processes are based on one, well characterized strain and can be repeated relatively well, cell and gene therapy manufacturing processes require increasingly adaptive process strategies that take the inherent variability of the living product into account. This can be achieved through a tight control over product quality attributes that can only be reached through an increased level of control over process parameters. Extensive online process monitoring and integrated control are required as they provide a crucial tool for process characterization and for detection and adaptation to process changes (Cierpka et al. 2013). However, basic knowledge of links between various parameters and process outcomes is often missing as it is difficult to define what needs to be measured and when. In addition, kinetics and balances in such complex biological systems are difficult to determine and describe. This inevitably makes control, reproducibility and repeatability of this kind of bioprocesses challenging. In this respect, better process monitoring could accelerate process development and improve production efficiency, while ensuring high-quality endpoint products (Rodrigues et al. 2011). Furthermore, documentation of process data is necessary to obtain regulatory approval (Cierpka et al. 2013) and approved release criteria.

Traditionally, post processing quality control (QC) is applied in the pharmaceutical industry to verify if the final product meets the set quality standards. However, when applied to the cell and gene therapy industry, this approach can be inadequate as these products encompass an inherent higher level of risk and variability whilst also being indication specific. A quality by design approach (QbD) would be more appropriate for the manufacture of these complex products, especially when it is the cell itself that is the product (Lipsitz et al. 2016). To this end, the field is moving towards a higher level of process understanding with more sophisticated real-time monitoring and control as well as the incorporation of advanced automated systems.

## The added value of automation

Automation can provide more control over a bioprocess through the use of sensors that produce online and continuous measurements, while leading to a more accurate and faster process optimization. Besides biological variation which is difficult to tackle due to the complexity of these products, in-process variation occurring from human handling is another persistent issue that can impact product quality. Even when stringent protocols are used, variation is observed between different handlers as a result of minor imprecisions in protocols (e.g. slight deviations in incubation times, variation in pipetting etc.). Automation can eliminate in-process variation through the use of robotic arms that can repeatedly and consistently perform a pipetting or a mixing action or even a whole cell culture sub-process (e.g. medium change) with consistent speed, force and accuracy, thus leading to reduced variability and increased process reliability.

Process parameters, settings and timing can be exactly determined, saved and tracked. Moreover, integration and automation of process analytics can eliminate the subjectivity of the judgments on which processing decisions are currently based and thus more sophisticated processing rules are made possible. For example, development of pattern recognition and image processing software can be used to objectively determine confluence. Cell culture protocols rely on passaging cells when they reach a confluence level of 70–80%. Confluency is typically estimated through microscopic visualisation of the cell culture and the percentage estimation is entirely subjective and dependent on the operator. The employment of automated image acquisition and processing can lay the foundation for adaptive processing based on objective and comprehensible criteria (Schenk et al. 2015). Additionally, automation can be adapted for increased throughput/parallelization of systems and it offers an enhanced possibility to monitor and track data.

In the cell and gene therapy realm, the benefits of automation could be translated into closed manufacturing platforms. The existing platforms will be discussed in the next sections where they are categorised depending on the level of automation integrated. Automation can refer to many approaches: automation of one step alone, integration of several steps in one machine (1st generation) or fully automated (2nd generation). It has to be noted here that the term “fully automated” refers to a platform or process which apart from eliminating manual operators for culturing cells, it also eliminates the need for manual transfer of materials from one-unit operation to another.

## Evolution of automation: from past to future

### 1st generation automated platforms

The 1st generation of automated systems came to address the lack of consistency due to manual handling. They entail the use of robotic arms and pipetting robots, programmed to imitate human actions, thus rendering the culturing of cells, more accurate, reproducible and consistent, while offering the possibility of processing larger volumes through a scaled-out approach. The CompacT SelecT™ (Sartorius) is one such system that makes use of an incubator, robotic arm and peristaltic pumps to carry out the different steps for cell culture. The CompacT SelecT has been used in commercial and academic settings for the subculture and expansion of different cell types in T-flasks (Soares et al. 2014; Thomas et al. 2009).

The Cellmate system (Sartorius) is another example that operates under the same principle allowing for higher volume expansion of cells in roller bottles and T-flasks without process change. This system is currently used by ReNeuron for the larger scale batch manufacture of their CTX stem cell therapies for stroke, as they move to phase III clinical trials (“ReNeuron to use Cellmate automated cell culture for stem cell-based stroke therapy—Cambridge Network” 2015). Avigen is another company that chose the Cellmate platform for manufacturing their adeno-associated viral vectored gene therapy product for clinical trials (“Cellmate—System Overview” n.d.).

More recently, numerous pipetting and liquid handling automated systems have emerged for the automation of laborious tasks, possibility to scale down or to increase the throughput. Examples include CyBio® (Analytik Jena), RoboLector (M2P Labs) (Kensy et al. 2009), Cello™ robot (Sartorius), Biomek® 4000 (Beckman), Freedom EVO (Tecan), STAR system (Hamilton), SimCell™. (Lindgren et al. 2009; Warr et al. 2009) These systems have been used successfully for cell culture applications (US Food and Drug Administration 2017), cell line development (Deng et al. 2011; Lindgren et al. 2009), cell characterisation or even for nanoscale assay development (Mosquito® - TTP Labtech) (Goadsby et al. 2017). However none of these systems are fully automated platforms capable of supporting a bioprocess from start to finish.

Another platform that was developed under the same principle is BioLector (M2P Labs) which provides a micro-fermentation system able to continuously monitor growth and fluorescence of recombinant reporter proteins under defined conditions in microtiter plates, incorporating online integrated analytics. This platform is suitable for downscaling and process development. However, there were no reports of using this platform with human cells, but only with bacterial or yeast cells (Back et al. 2016; Funke et al. 2010).

While the aforementioned platforms are suited for automated plate-based processes in high-throughput, they have limited applicability for cell and gene therapy production due their poor scalability and transferability to stirred tank systems. Consequently, platforms such as the Ambr15® and Ambr250® (Sartorius) revolutionized process development by introducing high-throughput options for cell culture, while using single use, disposable bioreactor vessels in an automated processing setting using an automated liquid handler. These platforms have been proven very useful for scale down studies and process development (Ryder et al. 2016). Both platforms make use of disposable pH and DO sensors, allowing for a better process control and facilitating scale-up of processes. Moreover, platforms such as these have been developed and adapted for culture of both suspension and adherent cells when grown on microcarriers (Nienow et al. 2013; Rafiq et al. 2017).

All of these systems come with advantages and disadvantages (Table 1). For example, systems such as the Ambr15 (Sartorius), STAR (Hamilton) and the Freedom EVO (Tecan) only accept manufacturer’s compatible pipette tips and specifically designed vessels, thus limiting their flexibility. Other limitations are related to the system’s functionality. For example, the STAR system (Hamilton) is only able to pipette small volumes at a time (5 mL), while the Ambr15 system is limited by the minimum agitation speed that can be employed (and hence minimum local energy dissipation rates), thus limiting its applicability. The CompacT SelecT (Sartorius), although suitable for adherent cell culture does not incorporate a centrifuge, thus the centrifugation step required for subculturing cells has to be performed outside the platform.

**Table 1:** Advantages and disadvantages of 1st generation automated cell culture systems

Culture system	Manufacturer	Advantages	Limitations
Freedom EVO	Tecan	– High precision – Allows for effective speeding up of processing – Offers liquid detection and notification for particles obstructing the pipetting	– Requires specific consumables – Requires special training for its programming and utilising fully the software’s capabilities
STAR	Hamilton	– High precision pipetting at small volumes – Modular design allowing for expansion as needs grow	– Requires specific consumables – Handles small volumes (< 5 mL) at a time
CompacT SelecT	Sartorius	– Suitable for both adherent and suspension cell culture – Ability to process up to 90 T175 flasks and 384 well plates – Runs subculture, cell counting and harvesting	– Requires additional pieces of equipment (e.g. centrifuge, microscope) to carry out the workflow – Large footprint
Biomek® 4000	Beckman Coulter	– Provides accuracy at handling small volumes	– Requires specific consumables
RoboLector	M2P labs	– Includes preparation of media – Allows for pH adjustments	– Volumes higher than 950 µL are pipetted in 2 steps
Cellmate	TAP Biosystems	– Using both flasks and roller bottles	– Does not incorporate automated harvesting
CyBio®	Analytik Jena	– Full assay automation including preparation of assay plates and measurements, cell seeding and incubator for further culture	– Only takes microplates – Requires specific consumables
Ambr15® and Ambr250®	Sartorius	– Proven scale down models – High throughput – Ability to run multiple conditions simultaneously – Suitable for optimisation studies	– Require specific consumables – Limited agitation speed range

In general, most of these liquid handling robots have large footprints, are expensive and require high servicing and maintenance costs. Additionally, they lack flexibility and they rely on additional pieces of equipment (e.g. centrifuge, incubator etc.) to carry out the workflow. They often require manufacturer specific consumables and their performance is highly dependent on the operator’s programming skills. However, robotic platforms can prove to be very useful for cell culture applications as they allow robustness and reliability and they minimise process variability by limiting the human error.

### 2nd generation automated platforms

Unlike the 1st generation, the 2nd generation of automated systems will allow for reduced manual handling as they offer complete automation on a sequence of operational units instead of only one. For example, these platforms would have the capability to receive donor tissue at one end and to offer a ‘polished product’ ready for distribution at the other end. These platforms will provide continuous process validation and monitoring which could enable better process understanding and faster optimisation. They would be fully closed integrated platforms, thus eliminating human contact with the source material or the cell product during processing. This would de-risk the production process by eliminating contamination, human error and would simplify the process by most likely rendering the use of clean rooms obsolete, thus minimising overall manufacturing costs. These platforms would be fully integrated, yet modular, allowing for flexibility which is key to the ever-evolving field of cell and gene therapy manufacturing.

An example of such a platform is represented by the CliniMACS Prodigy system (Miltenyi Biotech) that is a commercially available, fully integrated platform dedicated to autologous cell and gene therapy manufacturing. This platform allows for cell activation, transduction, amplification and final harvesting through unit operations such as cell enrichment via optimisation of cell surface markers, centrifugation and cultivation, all performed in one device. The CliniMACS Prodigy platform is equipped with integrated IPC/QC sampling pouches that allow the option to sample without opening the system by disconnection through sterile welding. Customisation of protocols is achieved through modularity and flexible programming, thus permitting its use for a variety of different cell types from the expansion of CAR-T cells, virus-specific T-cells, macrophages through to dendritic cells (Fraser et al. 2017; Mock et al. 2016; Zhu et al. 2016, 2019). For these reasons, the CliniMACS Prodigy platform was approved by the European Medicinal Agency (EMA) for the commercial manufacturing process of an already approved therapy e.g. Zalmoxis (MolMed) (Gladbach et al. 2018). However, this platform is only applicable to suspension cells and has not been tested to date for its suitability on isolating and expanding adherent cells. To this extent, it is limited in its applicability and not highly versatile. Additionally, this platform is limited to a patient-specific (i.e. autologous) approach, mainly due to the processing volumes of up to 400 mL. To develop an allogeneic therapy, multiple such platforms would be required to run in parallel through a scale-out rather than scale-up approach. Lastly, this all-in-one approach may create significant manufacturing issues as although the transduction step is performed in a matter of hours, the cell expansion requires many days, thus creating a bottleneck in manufacture by limiting the use of the platform to one patient at a time and for a significant period of time, inevitably resulting in high manufacturing costs.

Similarly, another ‘device-based’ production platform targeting autologous cell therapies is the Cocoon™ (Octane Biotech Inc.) which is an all-in-one, closed, non-agitated, fully automated platform that enables several operational units to be performed within a single chamber. The capabilities of this platform are: cell seeding, expansion, perfusion, digesting/harvesting, concentration, washing and formulation. However, similarly to the CliniMACS Prodigy, this platform is also limited to the CAR-T cell space and has not been tested for adherent cell culture (Iyer et al. 2018).

Another closed, automated system tested for both adherent and suspension cell culture is the Quantum™ (Terumo BCT). This platform is essentially a hollow fibre bioreactor that contains single-use cartridges, providing a surface area of 2.1 m^2^ per cartridge (Iyer et al. 2018). The Quantum™ has been successfully tested with adipose derived stromal cells (ASCs), bone marrow derived mesenchymal stromal cells (BMMSC) and neural stem cells (Haack-Sørensen et al. 2016; Martin-Manso and Hanley 2005). The Quantum only comprises of a bioreactor which limits its use to cell expansion only. This platform is not versatile enough to allow for other bioprocess enhancements such as cell enrichment or isolation from donor tissue.

More recently, through European research initiatives, a series of fully automated platforms capable of supporting the bioprocess from start to finish were developed. One such example is the Stem Cell Factory (Marx et al. 2013) that is a fully automated production unit for reprogramming, cultivation and differentiation of induced pluripotent stem cells (iPSCs). Its capability is up to 60 different iPSC lines in parallel.

**Fig. 1:**
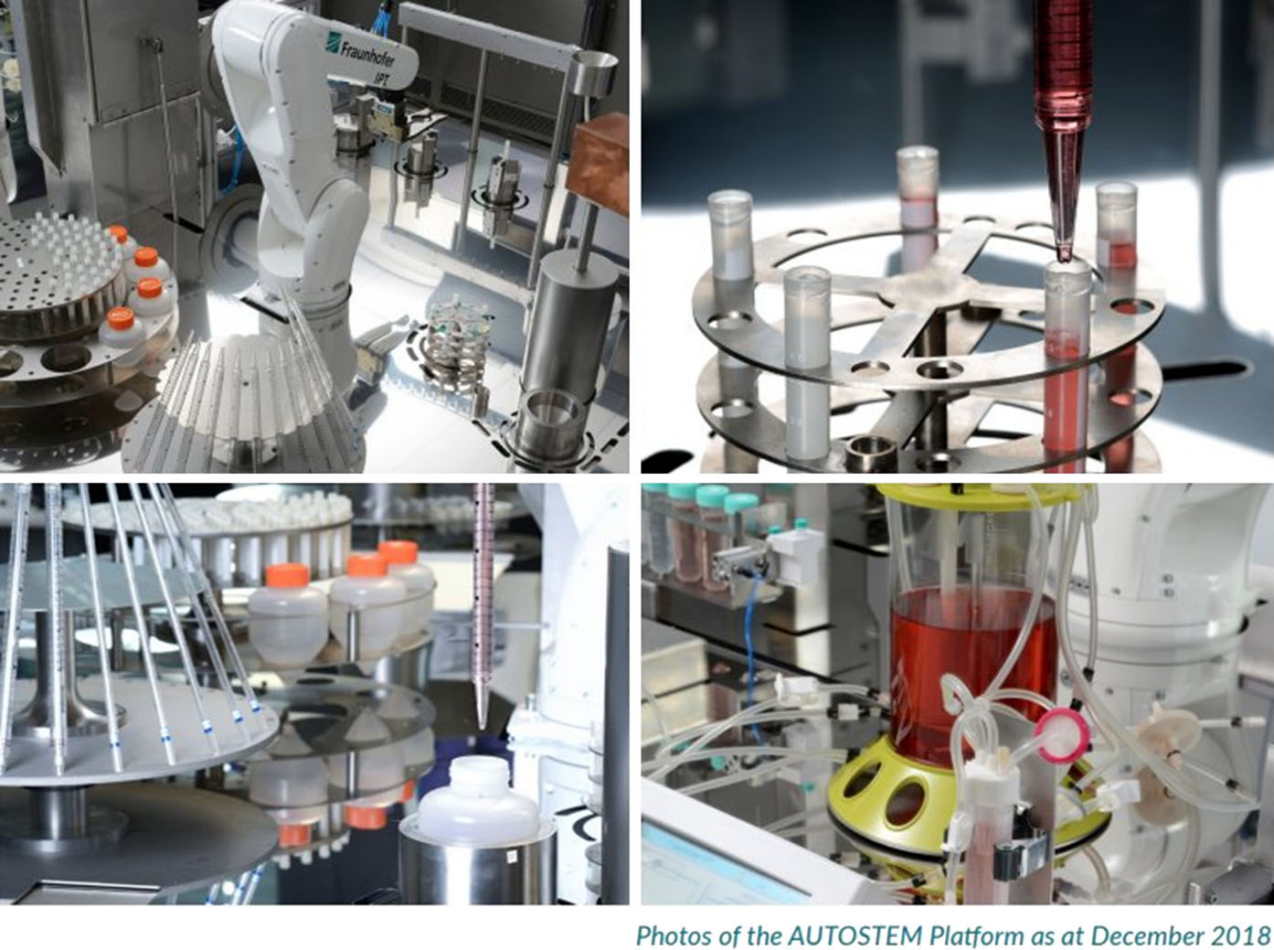
The AUTOSTEM platform

However, one versatile platform recently developed that would allow for a fully automated manufacturing and banking of cell therapies undertaking a donor-to-patient approach is the AUTOSTEM fully automated platform (Fig. 1). AUTOSTEM allows for tissue collection, isolation, cell expansion, harvest, concentration and cryopreservation of cell-based products. It is a fully enclosed, GMP-ready platform that comprises of a pipettor, robotic arms and proprietary technology in the unique design of grippers that allows versatility and flexibility in handling a variety of culture vessels (e.g. tubes, cryovials) for the different steps of the process. The AUTOSTEM platform was tested with human mesenchymal stem/stromal cells, however its modularity and flexibility allows for adaptation to other adherent cell types and even suspension cells, thus expanding its applicability and versatility (Callens et al. 2016; Murphy et al. 2017; Ochs et al. 2017).

These platforms offer the equivalent of a GMP manufacturing environment and are relevant for bench to bedside applications. The fully closed environment that these platforms provide is equivalent to a clean room environment, while the automation (in the form of robotic arms and control units) is equivalent or better than the highly trained personnel employed in GMP facilities. The GMP certified equipment remains the same, but it is now enclosed within a Class II cabinet using an automated platform and with LOG files produced and stored electronically during the process which forms the basis of the QM documentation required for every GMP manufacturing process.

Even if still under development, both the AUTOSTEM and the Cocoon systems offer more versatility than the existing platforms as they incorporate advanced bioreactor and scaffold technology coupled with complete automation of the different operational units required, with applicability in both adherent and suspension cell culture. Both systems receive patient tissue and perform the isolation of the targeted cells, followed by their expansion and quality control, all inside the enclosed platform, delivering cryovials of frozen cells or a living scaffold implant at the end of the process, while only requiring minimum manual handling for the loading of the consumables to the platform. In the case of the AUTOSTEM platform, an additional advantage is that loading of the tissue sample (i.e. the bone marrow) is also performed automatically as the bone marrow suction device is integrated into the platform. Analytics in the form of multiple capability sensors can also be integrated, further minimising the risk of contamination and improving culture monitoring capabilities (Murphy et al. 2017).

Another important advantage that these 2nd generation automated cell manufacturing platforms introduce to the field is the increased flexibility and modularity. Hardware modules are integrated in the platform via agents into the control software using a plug-and-produce approach and software that is adaptable to different applications (i.e. different process conditions or different cells). This is important as the field is continually evolving and the added complexity of bioprocesses using living cells will always be there, as biological processes are far from being understood or predicted to the same extent as chemical processes currently are (Jung et al. 2018).

## Conclusions and outlook

While the advanced therapy manufacturing is already profiting from automation approaches that have been used successfully in the ‘traditional’ production industries for a long time, it does not yet fully exhaust the potential of advanced manufacturing approaches which are currently being developed by the industry 4.0 (Kulik et al. 2016).

Automation and enabling technologies such as digitalisation, process analytical technologies and data processing have the potential to change the way we develop and produce cell and gene therapies. These new fully automated platforms (2nd generation) will provide a better understanding of the impact that processes might have on cell quality, while delivering enhanced reproducibility, facilitate regulatory compliance and lower manufacturing costs through optimised bioprocesses. By removing the manual handling completely, the safety and efficacy of the cell-based products will be enhanced. In the long term, automation can be the enabler of cost reduction, paving the way towards a broad application of cell and gene therapies as first-option treatments, while offering better accessibility for the patients.

To take this further, the next generation of automated cell manufacturing systems could potentially include artificial intelligence and machine learning tools. These could prove very helpful for process optimization and could minimize the challenges imposed by biological variability by revealing patterns and correlations between certain biological characteristics and process outcomes. This could potentially pave the way to a different approach for autologous therapies; for example, identifying donor material as a critical control point during the bioprocess and by incorporating a quality control step on the harvested tissue could help to further de-risk the process by making predictions on the outcome and effectiveness of the bioprocess.
